# Shensong Yangxin Protects Against Metabolic Syndrome-Induced Ventricular Arrhythmias by Inhibiting Electrical Remodeling

**DOI:** 10.3389/fphar.2020.00993

**Published:** 2020-07-09

**Authors:** Hong-Jie Yang, Bin Kong, Wei Shuai, Jing-jing Zhang, He Huang

**Affiliations:** ^1^ Department of Cardiology, Renmin Hospital of Wuhan University, Wuhan, China; ^2^ Cardiovascular Research Institute, Wuhan University, Wuhan, China; ^3^ Hubei Key Laboratory of Cardiology, Wuhan, China

**Keywords:** Shensong Yangxin, ventricular arrhythmia, electrical remodeling, ion current, gap junction

## Abstract

Shensong Yangxin (SSYX) is a traditional Chinese medicine, which has been proven to improve the clinical symptoms of arrhythmia. However, the role of SSYX in metabolic syndrome (MetS)-induced electrical remodeling remains to be fully elucidated. Here, we sought to clarify whether SSYX can alter the electrophysiological remodeling of cardiac myocytes from MetS rats by regulating transient outward potassium current (*I*_to_) and calcium current (*I*_Ca-L_). Male Wistar rats were subjected to 16 weeks of high-carbohydrate, high-fat to produce a MetS model group. SSYX (0.4 g/kg) was administrated by daily gavage 8 weeks following high-carbohydrate, high-fat for 8 weeks. *In vivo* electrophysiological study was performed to evaluated ventricular arrhythmias (VA) vulnerability and electrophysiological properties. The potential electrical mechanisms were estimated by whole-cell patch-clamp and molecular analysis. The H9C2 cells were used to verify the protective role of SSYX *in vitro*. After 16-week high-carbohydrate, high-fat feeding, MetS model rats showed increased body weight (BW), blood pressure (BP), blood sugar (BS), heart rate (HR) and heart weights to tibia length (HW/TL) ratio. Furthermore, MetS rats depicted increased VA inducibility, shortened effective refractory period (ERP) and prolonged action potential duration (APD). Lower *I*_Ca-L_ and *I*_to_ current densities were observed in MetS rats than CTL rats. Additionally, MetS rats exhibited significantly increased cardiac fibrosis, decreased Cx43 expression and protein levels of Cav1.2, Kv4.2, Kv4.3 than CTL group. As expected, these MetS-induced effects above were reversed when SSYX was administrated. Mechanistically, SSYX administrated significantly down-regulated the TLR4/MyD88/CaMKII signaling pathway both *in vivo* and *in vitro*. Collectively, our data indicated that the electrical remodeling induced by MetS contributed to the increased VA susceptibility. SSYX protects against MetS-induced VA by inhibiting electrical remodeling through TLR4/MyD88/CaMKII signaling pathway.

## Introduction

The term metabolic syndrome (MetS), which refers to a collection of abnormalities, including central obesity, hypertension, type 2 diabetes, and dyslipidemia (Liese et al., 1998; Hanci et al., 2011; Angeloni et al., 2012). Coronary heart disease (CHD) and total mortality are significantly higher in US adults with than in those without MetS (Malik et al., 2004). Previous studies reported that MetS can increases the risk of arrhythmia. Patients with MetS showed significantly risk of atrial fibrillation (Nyström et al., 2015; Kim et al., 2018). It has been demonstrated that MetS increase the risk of ventricular arrhythmia (VA) (Fernández-Sada et al., 2017; Liptak et al., 2017). Furthermore, MetS was reported to contribute to higher recurrence rate of outflow tract VA after catheter ablation (Sardu et al., 2014).

Previous studies demonstrated that electrical and structural remodeling of the ventricle, are suggested to be the basic mechanism of VA triggering and maintenance. Structural remodeling includes ventricular fibrosis (Brilla et al., 1992; Burchfield et al., 2013) and electrical remodeling manifest as shortened ERP, prolonged APD, ion channels remodeling and gap junction remodeling (Wasson et al., 2004; Yang and Nerbonne, 2016). Emerging evidence suggest that ventricular remodeling including electrical and structural remodeling contributing to MetS-induced VA (Lee et al., 2017; Durak et al., 2018). Theoretically, treatment targeting the ventricular remodeling would be of great benefit to the therapy of MetS-induced VA.

The traditional Chinese medicine (TCM) Shensong Yangxin (SSYX) including 12 medicinal materials (Ginseng, Ophiopogonis, *Cornus officinalis*, *Salvia miltiorrhiza*, *Ziziphi spinosae* semen, *Taxilli herba*, Paeoniae Radix Rubra, Eupolyphaga seu steleophaga, Nardostachyos, *Coptis chinensis*, *Schisandrae sphenantherae fructus*, and *Os draconis*) (Liu et al., 2015). The SSYX capsule has been widely applied to arrhythmia patients including ventricular premature complexes (VPC) and atrial premature complexes (APC) in China (Zou et al., 2011; Wang et al., 2017), implying that SSYX has anti-arrhythmic effect. However, whether SSYX has effect on MetS-induced VA, and the precise mechanisms remain unknown. Network pharmacology analysis is an excellent tool which has been widely used to analysis the interrelationship between TCM targets and disease targets and the underlying mechanisms (Chen et al., 2016). The current study tried to apply pharmacological network to analysis the potential targets and pathways of SSYX on MetS-induced VA. Moreover, animal experiments were also designed to check the role of SSYX on MetS-induced VA.

## Materials and Methods

### Animals

100 male Wistar rats (180–220 g) used in this study were provided by Renmin Hospital of Wuhan University’s animal experiment center. Rats were housed in conditions with temperature of 22 ± 2°C and a 12:12-h light/dark cycle, ad libitum food, and tap water. Rats were fed with normal diet (CTL) group or fed with 16-weeks of high-carbohydrate, high-fat diet (HCHF) including condensed milk (39.5%), beef tallow (20%), and fructose (17.5%) together with 25% fructose in drinking water to produce a MetS model group (Panchal et al., 2011). SSYX (0.4 g/kg) was administrated by daily gavage 8 weeks following HCHF diet for 8 weeks (Dang et al., 2016). SSYX used in this study were provided by Yiling Pharmaceutical Corporation (Shijiazhuang, China). Metoprolol (5 mg/kg) was administrated for 2 weeks before the end of MetS model by intraperitoneal injection once daily (Yang et al., 2019). Rats were weighed weekly. The serum glucose and blood pressure was measured after 16 weeks of HCHF diet. All experimental procedures were according to the guidance of Guidelines for the Care and Use of Laboratory Animals published by the US National Institutes of Health (NIH Publication No. 82-23, revised 1996) and were approved by Renmin Hospital of Wuhan University.

### Drugs

SSYX (batch number J111202) was provided by Yiling Pharmaceutical Corporation (Shijiazhuang, China) and the details of the contents and the voucher numbers of SSYX are shown in **Supplementary Table S1**. In 2014, through high-performance liquid chromatography coupled with electrospray tandem mass spectrometry (HPLC-ESI-MS/MS) method, Liu et al. (2014) identified ten major bioactive components (Morroniside, Loganin, Spinosin, Paeoniflorin, Coptisine, Epiberberine, Berberine, Palmatine, Schisantherin A, Deoxyschizandrin) of SSYX (batch number J111202) and the contents were 36.6, 53.8, 1.43, 24.6, 2.6, 1.47, 10.2, 7, 3.05, 2.8 ng/mL, respectively. Representative extracted ion chromatograms was shown in **Supplementary Figure S1**.

### *In Vivo* Electrophysiological Study

Rats were anesthetized (pentobarbital sodium, 40 mg/kg, i.p. Sigma) and then incubated and ventilated with a volume-constant rodent ventilator, and a left thoracotomy was performed. The heart was exteriorized from the thorax to prepare for the electrophysiological study.

### Action Potential Duration Measurement

The ventricular action potential duration (APD) measurement was performed with a S1S1 stimulation electrodes at pacing cycle lengths (CL) of 250, 200, 150, 100 ms with 10 stimulations. The APD was measured at 90% repolarization (APD90).

### Effective Refractory Period Measurement

The ventricular effective refractory period (ERP) was measured *via* an S1S2 stimulation protocol, 8 consecutive S1 stimulations with cycle length (CL) of 200 ms were followed by a stimulus S2, the CL was progressively reduced by 10 ms starting from 200 ms and ending at 20 ms, then reduced by 10 ms from 20 ms to the conduction block, and finally reduced in steps of 1 ms from the last conducted S2 to ERP (Yang et al., 2020).

### Ventricular Arrhythmias Inducibility

Burst pacing protocols were conducted to determine susceptibility to ventricular arrhythmias (VAs) as previously described (Yang et al., 2020). Briefly, VA was induced through the last 2-s burst pacing which repeated for three times. VAs were defined as consecutive premature ventricular contractions at least 2 s. And sustained VA were defined as the VA last more than 30 s. Susceptibility to VAs was evaluated based on the VA incidence and the ratio of sustained to nonsustained VAs (Yang et al., 2020).

### Isolation of Cardiomyocytes

Cardiomyocytes were isolated using previously described methods (Liu et al., 2017). In brief, rats were anesthetized by intraperitoneal injection of sodium pentobarbital (60 mg/kg). The hearts were excised rapidly and connected to a Langendroff apparatus with a constant flow at a rate of 8 to 10 mL/min for retrograde perfusion *via* the aorta, at 37°C for 5 min in Ca^2+^-free Tyrode’s solution containing with (in mmol/L): NaCl 130; KCl 5.4; MgCl_2_ 1; Na_2_HPO4 0.3; HEPES 10; glucose 10; PH adjusted to 7.35 with NaOH. The hearts were further digested with the same solution containing 0.3 mg/ml collagenase type II (Sigma, Co. US), 0.1% bovine serum albumin, and 30 μM CaCl_2_ for 15 to 20 min. At the end of the perfusion, the LV free wall was dissected from the heart and placed in cold KB solution (mM: taurine 10; glutamic acid 70; creatine 0.5; succinic acid 5; dextrose 10; KH_2_PO_4_ 10; KCl 20; HEPES 10; EGTA 0.2; PH adjusted to 7.35 with KOH). Cardiomyocytes were separated by pipetting, and calcium was reintroduced to suspend cells by gradual increases to a final concentration of 1 mM. The cardiomyocytes suspension was stored in KB solution at 4°C before Patch-Clamp recording.

### Whole Cell Patch-Clamp Recording

Whole-cell patch-clamp was performed using EPC-9 amplifier (List Instruments, Germany), and data were analyzed with Pulse-fit software interface (Version 8.31, HEKA Co. Germany). The resistances of the pipettes ranged from 3 to 6 MΩ when filled with pipette solution. Series resistance (Rs) was between 4 and 10 MΩ, and compensation was applied to reduce Rs by 80% to 90%. Current signals were filtered at 3 kHz by an eight-pole Bessel filter, digitized at a sampling rate of 1 kHz, stored on the computer running Pulse software which was used for the generation of pulses. Data analysis was used Clamp-fit 10.7 and Origin 9.0.

L-type calcium current (*I*_Ca-L_) was measured using the whole-cell voltage-clamp mode. The external solution contained (in mM): Choline chloride 100, NaCl 35, NaH_2_PO4 0.33 MgCl2 1, KCl 5.4, CaCl_2_ 1.8, HEPES 10, glucose 10, BaCl_2_ 0.1, 4-aminopyridine 5, and PH 7.4 with NaOH. The pipette solution contained (in mM): CsCl 120, EGTA 10, CaCl2 1, MgCl2 5, Na_2_-ATP 5, HEPES 10, and pH 7.2 with CsOH. The interval between pulses was 5 s. The whole-cell patch-clamp of *I*_Ca-L_ analyses included four parameters for each experimental group: (1) current-voltage relationship curves, (2) steady-state activation curves, (3) steady-state inactivation curves, and these data were also fitted to the Boltzmann distribution to obtain the half activation (V1/2)), (4) recovery curves from inactivation.

The pipette solution used to record transient outward potassium current (*I*_to_) using previously described methods (Liu et al., 2017) which contained (in mM): KCl 45, K-aspartate 85, Na-pyruvate 5, 5 mM MgATP 5, 10 mM EGTA 10, HEPES 10, and glucose 11 was adjusted to pH 7.35 with KOH. The external recording solution for *I*_to_ contained (in mM): choline chloride 110, NaCl 35, KCl 5.4, MgCl_2_ 1.0, NaH_2_PO_4_ 0.33, HEPES 10, and glucose 10 and was adjusted to pH 7.35 with NaOH. The whole-cell patch-clamp of *I*_to_ analyses included four parameters for each experimental group: (1) current-voltage relationship curves, (2) steady-state activation curves, (3) steady-state inactivation curves, and these data were also fitted to the Boltzmann distribution to obtain the half activation (V1/2)), (4) recovery curves from inactivation.

### Immunofluorescence

For immunofluorescence detection, the sections with a thickness of 10 μm were incubated with the primary antibody against Cx43 (1:200, Abcam, #ab79010), afterwards, sections were incubated with FITC-conjugated anti rabbit whole IgG and Texas Red-conjugated anti mouse whole IgG. Images were examined with a classic light microscope with epifluorescence equipment (CX-21, Olympus, Japan).

### Fibrosis Quantification

Isolated left ventricle (LV) were fixed in 10% phosphate-buffered formalin, and embedded in paraffin. Then the LV were sliced into 6-μm-thick sections. The picrosirius red (PSR) was performed to quantify cardiac fibrosis. The percentage of LV fibrosis was calculating by the ratio of LV fibrotic tissue area to normal LV area.

### Cell Culture and Treatment

H9C2 cells were obtained from the Cell Bank of the Chinese Academy of Sciences (Shanghai, China) and were cultured in DMEM medium, containing 10% fetal bovine serum (FBS, GIBCO, 10099) and 100 U/mL penicillin, at 37°C in a humidified atmosphere with 5% CO2. Then, H9C2 cells were seeded onto 6-well in DMEM with 10% FBS for 48 h. After that, the cells were starved for 16 h and then were infected with palmitate (PA, 50 mmol/L) and high glucose (HG, 25 mmol/L) or PBS for 24 h (Chan et al., 2019), then the H9C2 cells were infected with LPS (100 ng/ml), the TLR4 agonist or PBS for 24 h. After that, H9C2 cells were treated with SSYX (1.0 μg/ml) or PBS for 12 h (Liu et al., 2018).

### Western Blotting Analysis

Total protein was extracted and separated on SDS-PAGE (AS1086, ASPEN). Then proteins were transferred onto PVDF membranes (EMD Millipore, Billerica, MA). The membranes were incubated overnight at 4°C with the primary antibody of Cx43, Cav1.2, Kv4.2, Kv4.3, TLR4, MyD88, CaMKII, p-CaMKII (Thr287) and GAPDH. The total protein levels were normalized to GAPDH.

### Quantitative Real-Time PCR

Total LV RNA was prepared using TRIzol reagent (Invitrogen) from LV samples and cDNA was synthesized by reverse transcription with the PrimeScript RT reagent Kit (TaKaRa). Then, the quantitative real-time PCR (qRT-PCR) was performed with Applied Biosystems 7500 Fast qRT-PCR machine. The mRNA data were normalized to Gapdh. All primer details were listed as follows:

Collagen-I: forward: 5′-CCGTGACCTCAAGATGTGCC-3′ and reverse: 5′-GAACCTTCGCTTCCATACTCG-3′, Collagen-III: forward: 5′-GACCTCCTGGAAAAGATGGATC-3′ and reverse: 5′-AAATCCATTGGATCATCCCC-3′, TGF-β: forward: 5′-GTGGCTGAACCAAGGAGACG-3′ and reverse: 5′-AGGTGTTGAGCCCTTTCCAG-3′.

GAPDH: forward: 5′-CGCTAACATCAAATGGGGTG-3′ and reverse: 5′- TTGCTGACAATCTTGAGGGAG-3′ The mRNA data were normalized to Gapdh.

### Network Pharmacology-Based Analysis

#### Collection of Drug Targets for SSYX and Gene Targets for VA

The SymMap (https://www.symmap.org) database was used to identify the potential target of SSYX with the “*P* < 0.05” setting (Wu et al., 2019). The GeneCards database (http://www.genecards.org/) was used to identify the VA-related targets using the phrase “ventricular arrhythmia” as a keyword (Guo et al., 2020).

#### Construction of Networks and Analysis

String database (https://string-db.org) was used to analysis the interrelationship between SSYX-related targets and VA-related targets. The potential targets of SSYX and the potential targets of VA were uploaded to the String database and then selected the species option as “Homo sapiens” (von Mering et al., 2003). A Venn analysis was performed by introducing the obtained targets into the online website (Funrich, http://www.funrich.org/) to overlap the VA-related targets and SSYX-related targets (Pathan et al., 2015). String online database was performed to construct the protein-protein interaction (PPI) network (PPI combined score > 0.7). Cytoscape 3.5.1 was used to visualize and analysis the interaction of target-teaget network, the quantitative property “degree” of a node that refers to the number of edges linked to it, which suggested the importance of that node in the network (Smoot et al., 2011).

### Kyoto Encyclopedia of Genes and Genomes Pathway Enrichment Analysis for VA-Related Targets of SSYX

DAVID Bioinformatics Resources 6.7 (http://david.abcc.ncifcrf.gov/) (Kanehisa and Goto, 2000) was used to performed the Kyoto encyclopedia of genes and genomes (KEGG) pathway enrichment analysis and explore the underlying involved pathways of SSYX on VA. Then, R software was performed to run the ClusterProfiler package at a significance threshold of adjust *P* value < 0.05.

### Statistical Analysis

All data in the tables and figures are expressed as mean ± SEM and were analyzed using GraphPad Prism 7.0 software. Differences between two groups were assessed using Student’s t-test or chi-square test. The differences between multiple groups were analyzed with one-way analysis of variance (ANOVA). P < 0.05 was considered as statistically significant.

## Results

### Systemic Effects of SSYX Administration in MetS Rats

**Figure 1:**
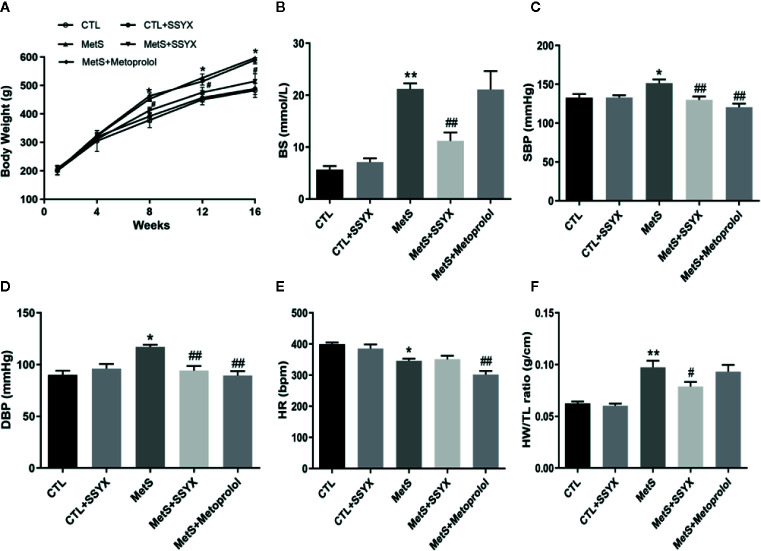
Systemic effects of SSYX administration in MetS rats. **(A)** Body weight, **(B)** BS, **(C)** SBP, **(D)** DBP levels, **(E)** HR and **(F)** heart weight to tibia length (HW/TL) ratio after 16-weeks of high-carbohydrate, high-fat diet. Data are expressed as the mean ± SEM. n = 5 per group. *P < 0.05 vs. CTL group. **P < 0.01 vs. CTL group. ^##^ *P* < 0.01 vs. MetS group.

At the end of the 16-week high-fat diet feeding, the body weights of MetS rats were significantly elevated compared with CTL group, and SSYX administration to MetS rats significantly reduced body weights compared with MetS rats (**Figure 1A**). As shown in **Figure 1B**, high-fat diet induced hyperglycemia were significantly reduced when SSYX were administrated, the β-blocker, metoprolol has no effect on body weights and BS in MetS rats. Furthermore, MetS rats exhibited both elevated systolic blood pressure (SBP) and diastolic blood pressure (DBP) compared with CTL group, and SSYX significantly lowered MetS-induced elevated blood pressure (**Figures 1C, D**), but the effect of blood pressure lowering of SSYX was less than the effect of metoprolol. Moreover, the SSYX administration has no effect on the lowered heart rate (HR), while metoprolol significantly reduce MetS-induced lowered HR (**Figure 1E**). In addition, MetS rats showed significantly increased heart weights to tibia length (HW/TL) ratio and SSYX significantly decreased MetS-induced HW/TL ratio (**Figure 1F**), however, metoprolol has no effect on HW/TL ratio in MetS rats. These results suggest that SSYX mitigate MetS-related metabolic disorder.

### Effect of SSYX Administration on VAs Vulnerability in MetS Rats

**Figure 2:**
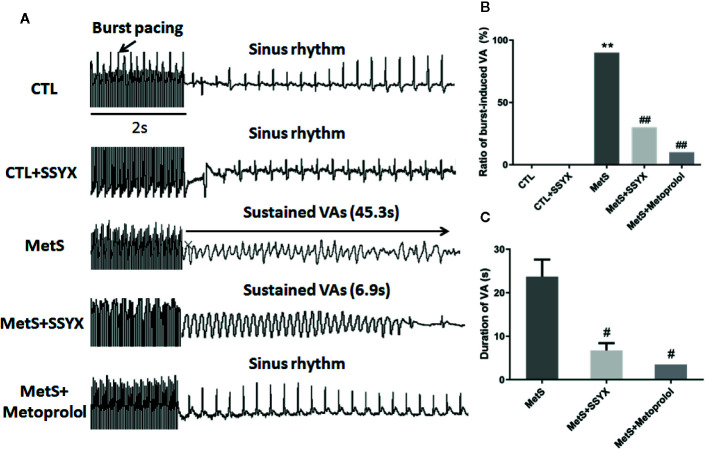
Effect of SSYX administration on VAs vulnerability in MetS rats **(A)** Representative examples of sinus rate (SR) and VA induced by burst stimulating and statistical analysis in the five groups (n = 10 per group). **(B)** Ratio of burst-induced VA and **(C)** duration of VA in the five groups (n = 10 per group). *P < 0.05 vs. CTL group. **P < 0.01 vs. CTL group. ^##^ *P* < 0.01 vs. MetS group.

Burst pacing was conducted to identify the role of SSYX on VA vulnerability induced by MetS. VA could not be induced in CTL group and CTL+SSYX group (**Figure 2A**). The VA induction ratio in MetS group was significantly increased when compared with CTL group (90% vs. 0%, *P* < 0.01, **Figure 2B**). And SSYX administrated significantly reduced VA susceptibility when compared with MetS group (90% vs. 30%, *P* < 0.01), while VA susceptibility was lower when metoprolol administrated compared to those administrated with SSYX (10% vs. 30%). Furthermore, SSYX administrated also significantly reduced duration of VA when compared with MetS group (6.8 ± 1.6 vs. 23.7 ± 3.9 s, *P* < 0.05, **Figure 2C**). These results manifested that MetS increased VA inducibility and SSYX protected against MetS-induced VA.

### Effects of SSYX Administration on the Electrophysiological Properties in MetS Rats

**Figure 3:**
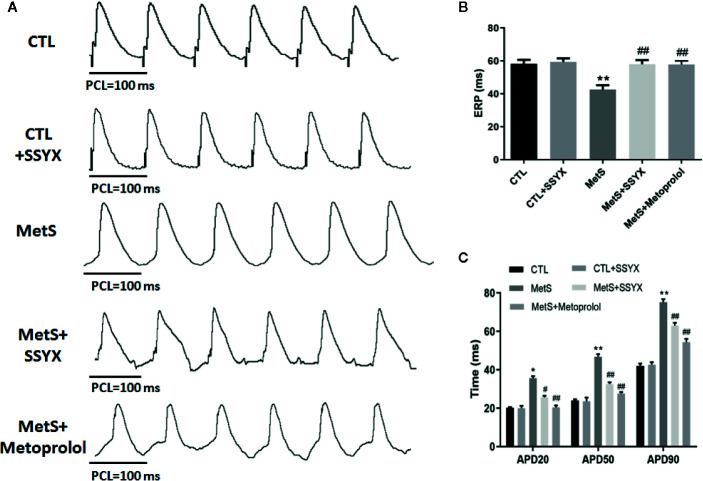
Effects of SSYX administration on the electrophysiological properties in MetS rats **(A)** Representative examples of APD. **(B)** Statistical analysis of ERP and **(C)** APD in the five groups (n = 10 per group). *P < 0.05 vs. CTL group. **P < 0.01 vs. CTL group. ^#^ *P* < 0.05 vs. MetS group. ^##^ *P* < 0.01 vs. MetS group.

The characteristics of left ventricular electrophysiological remodeling were analyzed in *in vivo* hearts. There was no difference in ERP between CTL group and CTL+SSYX group (*P* > 0.05). Compared with the CTL group, the MetS rats displayed a significant shorten in ERP (42.6 ± 2.6 vs. 58.2 ± 2.4 ms; *P* < 0.01, **Figure 3B**), while SSYX reversed the change induced by MetS (58.0 ± 2.5 vs. 42.6 ± 2.6 ms, *P* < 0.01, **Figure 3B**).

The effect of SSYX on the alterations of APD induced by MetS is shown in **Figure 3A**. There was no difference in APD_20_, APD_50_, APD_90_ between CTL group and CTL+SSYX group (*P* > 0.05). Compared with CTL group, the MetS rats displayed significantly prolonged APD_20_, APD_50_, APD_90_ (*P* < 0.01 for all, **Figure 3C**), while SSYX reversed the APD change induced by MetS (**Figure 3C**). Altogether, these data indicated that SSYX offset MetS-induced electrophysiological remodeling.

### SSYX Administration Affects the *I*_Ca-L_ Current in Cardiomyocytes From MetS Rats

**Figure 4:**
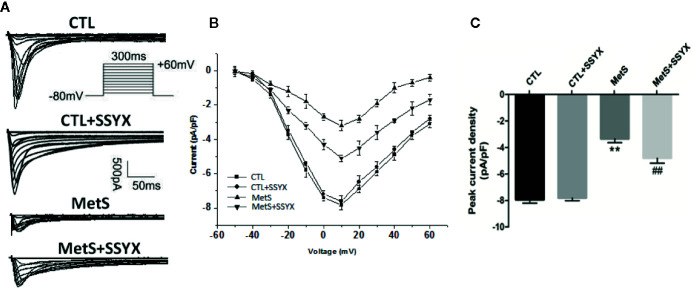
SSYX administration affects the *I*_Ca-L_ current in cardiomyocytes from MetS rats **(A)** Typical examples of L-type calcium current tracings. **(B)** Current density-voltage (I-V) correlation for L-type calcium current. **(C)** The maximum L-type calcium current density (n=8 cardiomyocytes from n=3 rats each group). **P < 0.01 vs. CTL group. ^##^ *P* < 0.01 vs. MetS group.

Previous studies reported that *I*_Ca-L_ current contribute to action potential (AP) repolarization. So we checked whether SSYX affects *I*_Ca-L_ current in cardiomyocytes from MetS rats. **Figure 4A** showed the representative *I*_Ca-L_ currents traces of the four group (**Figure 4A**). MetS myocytes showed significantly lower peak current density than the CTL group (−3.35 ± 0.29 vs. −7.94 ± 0.27 pA/pF, respectively; *P* < 0.01, **Figures 4B, C**), whereas peak *I*_Ca-L_ amplitude was significantly increased when SSYX was administrated in MetS rats than MetS group (−4.80 ± 0.38 vs. −3.35 ± 0.29 pA/pF, respectively; *P* < 0.01, **Figures 4B, C**). No significant peak *I*_Ca-L_ amplitude was found between CTL+SSYX myocytes and CTL myocytes (**Figure 4C**). We further detected the kinetic characteristics of *I*_Ca-L_. As depicted in **Table 1**, the inactivation curve of MetS myocytes were markedly right-shifted relative to the CTL group (V_1/2_: −15.5 ± 2.5 mv vs. −23.2 ± 1.6 mv, *P* < 0.05), and the inactivation curve of MetS+SSYX myocytes were markedly left-shifted relative to the MetS group (V_1/2_: −21.9 ± 1.2 mv vs. −15.5 ± 2.5 mv, *P* < 0.05). No significant differences were found between CTL and CTL+SSYX myocytes (*P* > 0.05). Besides, the half activation voltage and recovery time constant were no significantly in four groups (*P* > 0.05, respectively; **Table 1**).

**Table 1:** Effects of SSYX administration based on MetS rats on *I*_Ca-L_ channel kinetics.

	CTL	CTL+SSYX	MetS	MetS+SSYX
Activation V_1/2_ (mv)	−21.8 ± 1.9	−22.0 ± 2.3	−20.6 ± 1.5	−1.2 ± 1.8
Inactivation V_1/2_ (mv)	−23.2 ± 1.6	−24.1 ± 1.7	−15.5 ± 2.5^*^	−21.9 ± 1.2^#^
Recovery from inactivation τ (ms)	−72.6 ± 8.1	−80.1 ± 5.9	−89.5 ± 11.3	−86.7 ± 8.7

Values presented are mean± SEM (n = 8 cells/group), *p < 0.05 vs. CTL group, ^#^p < 0.05 vs. MetS group.

### SSYX Administration Affects the *I*_to_ Current in Cardiomyocytes from MetS Rats

**Figure 5:**
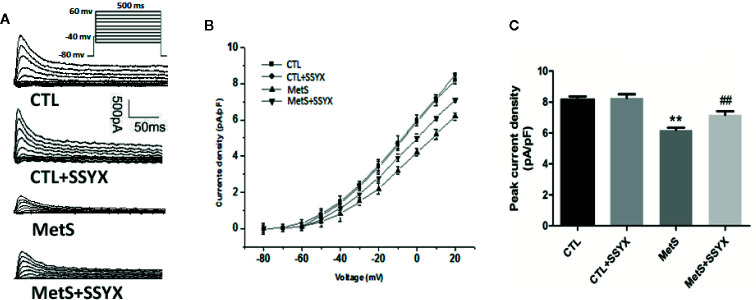
SSYX administration affects the *I*_to_ current in cardiomyocytes from MetS rats **(A)** Typical examples of *I*_to_ current tracings. **(B)** Current density-voltage (I-V) correlation for *I*_to_ current. **(C)** The maximum *I*_to_ current density (n=8 cardiomyocytes from n=3 rats each group). **P < 0.01 vs. CTL group. ^##^ *P* < 0.01 vs. MetS group.

*I*_to_ current is also important in contributing to phase 1 AP. Representative *I*_to_ currents traces of the 4 groups were exhibited in **Figure 5A**. MetS myocytes showed significantly lower peak current density than the CTL group (6.19 ± 0.16 vs. 8.16 ± 0.20 pA/pF, *P* < 0.01, **Figures 5B, C**), whereas peak *I*_to_ amplitude was significantly increased when SSYX was administrated in MetS rats than MetS myocytes (7.16 ± 0.24 vs. 6.19 ± 0.16 pA/pF, *P* < 0.01, **Figures 5B, C**). No significant peak *I*_to_ amplitude was found between CTL+SSYX myocytes and CTL myocytes (**Figure 5C**). We further detected the kinetic characteristics of *I*_to_. The *I*_to_ recovery time constant was significantly increased in the MetS myocytes compare to CTL myocytes (τ: −57.3 ± 1.5 vs. −36.5 ± 1.2, respectively; *P* < 0.01, **Table 2**), and MetS+SSYX myocytes significantly decreased *I*_to_ recovery time constant compare to MetS myocytes (τ: −43.7 ± 1.7 vs. −57.3 ± 1.5, *P* < 0.01). Besides, the half activation voltage and half inactivation voltage were no significantly in four groups (*P* > 0.05, respectively; **Table 2**).

**Table 2:** Effects of SSYX administration based on MetS rats on *I*_to_ channel kinetics.

	CTL	CTL+SSYX	MetS	MetS+SSYX
Activation V_1/2_ (mv)	−16.7 ± 2.1	−18.2 ± 1.8	−15.9 ± 1.7	−16.2 ± 1.3
Inactivation V_1/2_ (mv)	−31.1 ± 1.6	−29.1 ± 1.9	−27.5 ± 2.7	−28.6 ± 2.4
Recovery from inactivation τ (ms)	−36.5 ± 1.2	−35.8 ± 0.9	−57.3 ± 1.5^**^	−43.7 ± 1.7^##^

Values presented are mean± SEM (n=8 cells/group), **p < 0.01 vs. CTL group, ^##^p < 0.01 vs. MetS group.

### SSYX Administration Affects the Gap Junction in MetS Rats

**Figure 6:**
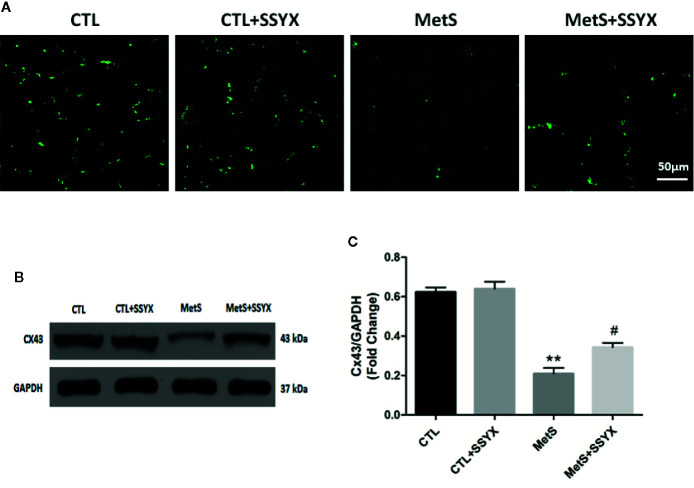
SSYX administration affects the gap junction in MetS rats **(A)** Typical examples of Cx43 immunofluorescence staining. **(B)** Representative Western blots and **(C)** quantitative results of the Cx43 (n = 3 per group). **P < 0.01 vs. CTL group. ^#^ *P* < 0.05 vs. MetS group.

Because arrhythmia propensity can be altered by gap junction remodeling, we then assessed gap junction remodeling by analyzing distribution of Cx43 with immunofluorescence staining. As shown in **Figure 6A**, immunosignal of Cx43 showed a significantly reduced in MetS group when compared with CTL group and SSYX administration in MetS rats significantly increased Cx43 immunosignal compared with MetS group. Additionally, the trend of Cx43 protein expression were consistent with the trend of Cx43 immunofluorescence signal. As shown in **Figures 6B, C**, the expression of Cx43 in MetS group were obviously decreased when compared to the CTL group (*P* < 0.01, **Figures 6B, C**), administration of SSYX in MetS rats significantly increased Cx43 expression compared with MetS group (*P* < 0.05, **Figures 6B, C**).

### SSYX Administration Affects the Cardiac Fibrosis in MetS Rats

**Figure 7:**
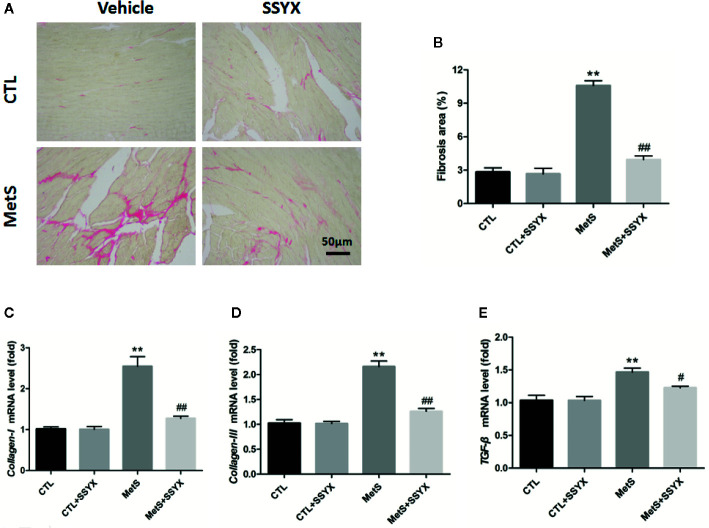
SSYX administration affects the cardiac fibrosis in MetS rats **(A)** Representative images of picrosirius red (PSR) stained heart sections and **(B)** quantitative results of fibrosis area. **(C–E)** Results of *collagen-I*., *III*, and *TGF-β* mRNA expression (n = 3 per group). **P < 0.01 vs. CTL group. ^#^ *P* < 0.05 vs. MetS group. ^##^ *P* < 0.01 vs. MetS group.

Because cardiac fibrosis is an important parameter for impulse conduction and VA, using picrosirius red (PSR) staining to quantify cardiac fibrosis. **Figure 7A** shows representative interstitial collagen among the four groups. Quantification revealed no significant differences in collagen content between CTL and CTL+SSYX hearts (*P* > 0.05, **Figure 7B**), after 16 weeks high-fat diet feeding, collagen content in MetS group significantly increased when compared with CTL group (*P* < 0.05, **Figure 7B**) and the fibrosis induced by MetS were ameliorated when SSYX was administrated (*P* < 0.05, **Figure 7B**). Additionally, *collagen-I*, *III*, and *TGF-β* mRNA expression in ventricle significantly increased in MetS group when compared with CTL group (*P* < 0.01, **Figures 7C–E**), and the change induced by MetS were ameliorated when SSYX was administrated (*P* < 0.01, **Figures 7C–E**).

### SSYX Administration Affects the Expression of Ion Channel Protein

**Figure 8:**
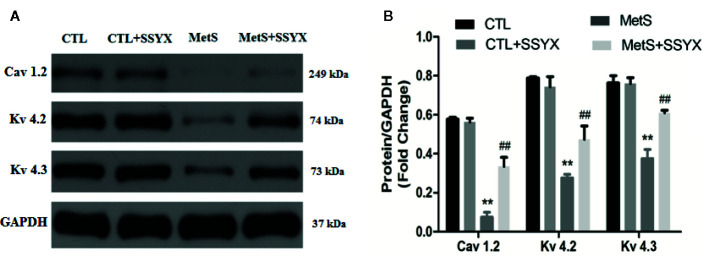
SSYX administration affects the expression of ion channel protein **(A)** Representative Western blots and **(B)** quantitative results of the Kv4.2, Kv4.3, and Cav1.2 expression (n = 3 per group). **P < 0.01 vs. CTL group. ^##^ *P* < 0.01 vs. MetS group.

Ion channels remodeling is reported to contribute to VA. We evaluated the protein expression levels of *I*_to_ and *I*_Ca-L_ channels in rat heart. The protein expressions of Kv4.2 and Kv4.3, which both encode *I*_to_ (Guo et al., 1999; Patel and Campbell, 2005), were significantly decreased in the MetS rats compared to CTL rats (*P* < 0.01, **Figure 8**). Administration of SSYX in MetS rats significantly increased the protein expressions of Kv4.2 and Kv4.3 compared to MetS rats (*P* < 0.01, **Figure 8**). The protein expression of Cav1.2, the pore-forming subunit of *I*_Ca-L_ (Fukuyama et al., 2014), was markedly reduced in the MetS hearts compared to CTL group (*P* < 0.01, **Figure 8**). Administration of SSYX in MetS rats significantly increased the protein expressions of Cav1.2 compared with MetS rats (*P* < 0.01, **Figure 8**). Above results demonstrated that SSYX could significantly up-regulate the expression of ion channel proteins in MetS hearts.

### Targets Network Analysis Relevant to the SSYX Treatment of MetS-Induced VA

**Figure 9:**
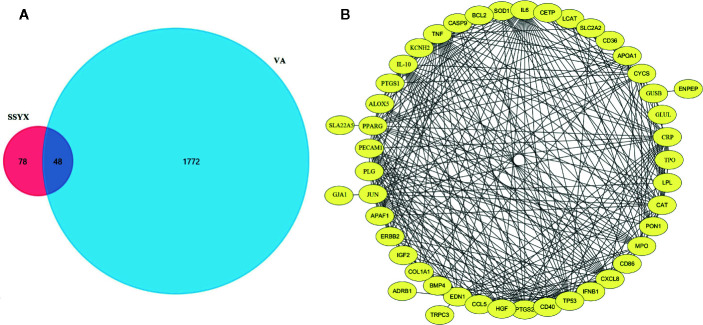
SSYX-VA target network **(A)** Venn diagram of SSYX and VA intersection targets. **(B)** Protein-protein interaction (PPI) network of targets of SSYX against MetS-induced VA.

As SSYX contains several herbs which depicted several pharmacological effects *via* multiplex targets, there is need to explore the underlying mechanism of SSYX on VA by targets network analysis. As shown in **Figure 9A**, according to the Venn diagram of the intersection of the SSYX targets and the VA targets, 48 coincidence targets (TRPC3, TPO, TP53, TNF, SOD1, SLC2A2, SLC22A5, SFTPB, PTGS2, PTGS1, PPARG, PON1, PLG, PECAM1, NR1I3, MPO, LPL, LCAT, JUN, IL6, IL10, IGF2, IFNB1, HGF, GUSB, GLUL, GJA1, ERBB2, ENPEP, EDN1, CYCS, CXCL8, CRP, COL1A1, KCNH2, CETP, CD86, CD40, CD36, CCL5, CAT, CASP9, BMP4, BCL2, APOA1, APAF1, ALOX5, ADRB1) were found. The PPI network targets of SSYX against MetS-induced VA was exhibited in **Figure 9B**, and we found that the numbers of nodes was 46, the number of interaction edges was 305.

### SSYX Administration Regulates the TLR4/MyD88/CaMKII Signaling Pathway

**Figure 10:**
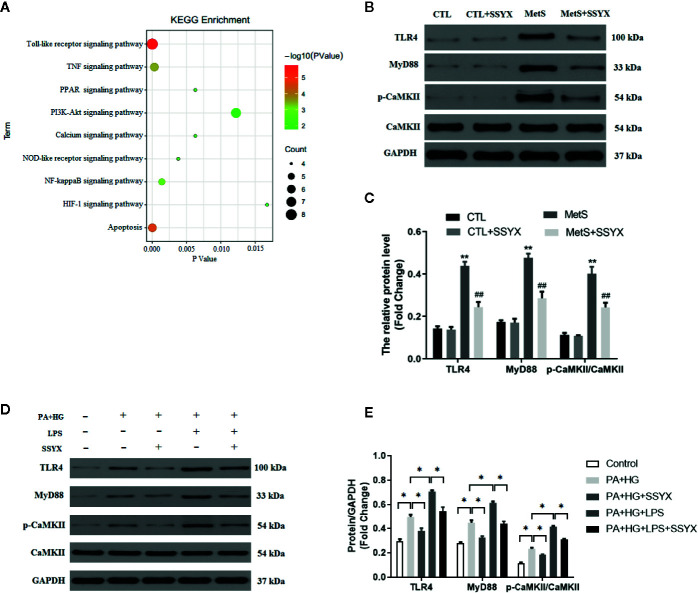
SSYX administration affects the TLR4/MyD88/CaMKII signaling pathway-related protein expression. **(A)** Bubble chart of top 9 KEGG signaling pathways linked to SSYX against MetS-induced VA. **(B)** Representative Western blots and **(C)** quantitative results of the TLR4, MyD88, CaMKII, and p-CaMKII expression (n = 3 per group). **(D)** Representative Western blots and **(E)** quantitative results of the TLR4, MyD88, CaMKII, and p-CaMKII expression in H9C2 cells (n = 3 per group). **P < 0.01 vs. CTL group. ^##^ *P* < 0.01 vs. MetS group. For **(D**, **E)**, *P < 0.05.

To find the underlying pathways of underlying the treatment effects of SSYX on MetS-induced VA, the KEGG pathway enrichment analysis of involved targets was conducted *via* the functional annotation tool of DAVID Bioinformatics Resources 6.7. The top 9 significantly enriched pathways of SSYX on VA were exhibited in **Figure 10A**. Among these candidate signaling pathways, the toll-like receptor (TLR) signaling pathway was identified as the relatively most significantly pathway. Furthermore, our former work demonstrated that TLR4/MyD88/CaMKII signaling pathway contributed to obesity-induced ventricular electrical remodeling (Shuai et al., 2019). So we tried to evaluate whether TLR4/MyD88/CaMKII signaling pathway involved in the protective role of SSYX against MetS-induced electrical remodeling. As shown in **Figures 10B, C**, the protein levels of TLR4, MyD88, and p-CaMKII were significantly up-regulated in MetS ventricle. And SSYX administrated significantly down-regulated the TLR4/MyD88/CaMKII signaling pathway induced by MetS, which further validated the role of TLR signaling pathway in the treatment effect of SSYX on MetS-induced VA. To further elucidate the effect of TLR4/MyD88/CaMKII signaling pathway in the pathogenesis of electrical remodeling induced by MetS, we treated the H9C2 cells with LPS, the TLR4 agonist. As shown in **Figures 10D, E**, the protein levels of TLR4, MyD88, and p-CaMKII were significantly up-regulated when PA+HG was treated and treated the H9C2 cells with PA+HG and LPS further increased TLR4, MyD88, and p-CaMKII protein levels, SSYX could significantly decreased these proteins induced by PA+HG or PA+HG and LPS. In consequence, SSYX protect against MetS-induced ventricular electrical remodeling may through TLR4/MyD88/CaMKII signaling pathway.

## Discussion

The present study mechanistically investigated the effect of SSYX on cardiac electrical remodeling and arrhythmogenesis in MetS rats. In this study, we provided evidences that SSYX treatment: 1) ameliorates MetS-induced VA susceptibility, shortened ERP and increased APD; 2) reverses MetS-induced downregulation of *I*_Ca-L_ and *I*_to_ current densities; 3) improves MetS-induced gap junction remodeling, cardiac fibrosis; 4) reverses MetS-induced downregulation of protein expression of Cav1.2, Kv4.2, Kv4.3; 5) and down-regulated the TLR4/MyD88/CaMKII signaling pathway induced by MetS. These findings hinted that SSYX could serve as a promising anti-arrhythmic agent by reversing electrical remodeling through regulating TLR4/MyD88/CaMKII signaling pathway induced by MetS.

The anti-arrhythmia effects of SSYX have been reported previously. Since the SSYX capsule was approved by the State Food and Drug Administration (SFDA) of China in 2003, SSYX has been widely used to treat the patients with VPC and APC in China (Zou et al., 2011; Wang et al., 2017). However, the precise mechanism remains unknown. Previous study reported that SSYX could reverse atrial electrical remodeling and the atrial fibrillation by adjusting the autonomic nerve activity and inhibiting atrial inflammation (Zhao et al., 2017). Another study reported that SSYX could reduce acute myocardial ischemia (AMI)-induced VA by inhibiting transient outward K^+^ current (*I*_to_) and reducing intracellular Ca^2+^ concentration of the cardiomyocytes (Zhao et al., 2016). Although SSYX may exert anti-arrhythmia effect by reversing electrical remodeling, until now, the role of SSYX in the setting of MetS-induced VA remains unclear. The present study uncovered that SSYX also protects against MetS-induced VA by inhibiting electrical remodeling.

Excessive APD prolongation and shortened ERP are hallmark of the abnormally altered electrophysiology or adverse electrical remodeling (Medei et al., 2008; Tse and Yan, 2017). These electrical remodeling may contribute to the conduction abnormalities and reentry, which is the m mechanism underlying the VA (Cherry et al., 2012). Durak et al. reported that prolonged APD_20_, APD_50_, APD_90_ was found in MetS rats (Durak et al., 2018). In the present study, MetS rats showed prolonged APD_20_, APD_50_, APD_90_ compared with CTL rats, SSYX significantly reduced MetS-induced prolonged APD. Additionally, SSYX also significantly increased MetS-induced shortened ERP. Taken together, SSYX reversed MetS-induced electrical remodeling by shortening APD and increased ERP. These data demonstrated that shortened ERP and prolonged APD may contribute to the electrical remodeling in MetS rats.

It is well-known that action potential (AP) due to kinds of transmembrane ions transmembrane movement. Abnormal of ion currents including K^+^ and Ca^2+^ channels, which contribute to the AP prolongation, electrical remodeling and lead to cardiac arrhythmias eventually. AP prolongation can be caused by decreased K^+^ currents, or increased Ca^2+^ currents, or both of them. Previous studies have shown that reducing the proteins of outward potassium channels could cause decreased outward potassium current density, eventually, induce a prolongation of the APD (Medei et al., 2010; Morrow et al., 2011). In this study, we found that the current densities and protein expression (Kv4.2, Kv4.3) of *I*_to_ were obviously decreased in MetS rats. Similar to our findings, Shuai et al. also found reduced protein expression of Kv4.2, Kv4.3 in high-fat diet mice (Shuai et al., 2019). Meanwhile, Grandinetti et al. reported that obese patients showed decreased *I*_to_ current densities, delay repolarization, and prolong APD (Grandinetti et al., 2010). In the present study, we tried to investigate whether SSYX has effect on *I*_to_ current, as expected, SSYX could increase *I*_to_ current and the protein expression of Kv4.2 and Kv4.3, which are similar to Zhao et al. reported. In consequence, these data suggest that depressed *I*_to_ current may contribute to the prolonged APD and lead to electrical remodeling eventually, SSYX may serve as an anti-arrhythmic agent through down-regulates the *I*_to_ current, protein levels of Kv4.2, Kv4.3.

*I*_Ca-L_ current also contribute to the AP prolongation by affecting phase 2 depolarization of action potential, reduced *I*_Ca-L_ current density may result in shortened AP. However, in the present study, we found reduced *I*_Ca-L_ current density accompanied with prolonged AP in MetS rats. It should take note that both inactivation and recovery from inactivation of *I*_Ca-L_ may affect the AP repolarization (Burger et al., 2009). In the present study, the kinetic characteristics of *I*_Ca-L_ showed the inactivation curve of MetS cardiomyocytes were markedly right-shifted relative to the CTL group. Taken all results together, we believe that reduced *I*_to_ current and interfered *I*_Ca-L_ inactivation may contribute to AP prolongation in MetS rats.

Gap junctions consist of connexin which contribute to electrical communication. Connexin43 (Cx43) is the major connexin protein in ventricular gap junction, which is critically linked to ventricular homogeneity of electric conduction and ventricular arrhythmias (van Rijen et al., 2004). Apart from ion current, gap junction also contribute to the electric remodeling and linked to VA. In this study, MetS rats showed reduced Cx43 immunofluorescence signal and protein expression. And SSYX significantly increased Cx43 protein levels.

The Venn diagram of the intersection of the SSYX targets and the VA targets, 48 coincidence targets, and the PPI network demonstrated that SSYX-related targets against VA including 46 nodes. Of the 46 genes, GJA1, COL1A1, and KCNH2 were identified to be related with our findings in the animal model. Previous studies reported that, GJA1 encoded Cx43 plays a critical role in intercellular communication between cardiomyocytes (Basheer et al., 2018), COL1A1 encoded collagen-I chains involved in ventricular arrhythmia (Yao et al., 2020), moreover, KCNH2 encoded the voltage-gated potassium channel, which is a critical current for the repolarization phase of the APD (Mazzanti et al., 2017). These targets network analysis further validated our findings that gap junction, ventricular fibrosis, and ion channels remodeling involved in the treatment effect of SSYX on VA.

The mechanisms underlying the protective effect of electrical remodeling induced by MetS remains to be elucidated. The KEGG pathway enrichment analysis revealed that the nine significantly pathways including toll-like receptor, TNF, PPAR, PI3K-Akt signaling pathway, Calcium signaling pathway, NOD-like receptor, NF-kappaB (NF-κB), HIF-1, and apoptosis signaling pathway, which involved in the treatment effect of SSYX on VA. Among these candidate signaling pathways, the toll-like receptor (TLR) signaling pathway was identified as the relatively most significantly pathway. Previous studies reported that TLR4/NF-κB signaling cascades contributes to VA under myocardial ischemia condition (Jiang et al., 2019; Wang et al., 2019). Our former work demonstrated that TLR4/MyD88/CaMKII signaling pathway contributed to obesity-induced ventricular electrical remodeling (Shuai et al., 2019). Previous studies demonstrated that TLR4 activation could active CaMKII cascades under myocardial stress and TLR4 is the upstream regulator of CaMKII, CaMKII exert a vital effect in obesity-induced electrical remodeling (Zhong et al., 2017). Activation of CaMKII can increase the L-type Ca^2+^ current, decrease the K^+^ current, and prolong APD (Li et al., 2006; Zhong et al., 2017). In addition, inhibited CaMKII with KN93 significantly reverse high-fat diet induced-electrical remodeling including increased Cx43 expression, decreased interstitial fibrosis, and increased Cav1.2, Kv4.2, Kv4.3 proteins expression (Zhong et al., 2017). Concluding these clues, TLR4/MyD88/CaMKII signaling cascades may contribute to the protective role in MetS-induced electrical remodeling. In line with previous reported, the western blot analysis further validate the role of TLR4/MyD88/CaMKII signaling pathway in the effect of SSYX on MetS-induced electrical remodeling.

A limitation of this study is that the active compounds in SSYX, which are responsible for the regulation of VA, remain unknown although it is well known that SSYX is a classical TCM which widely used for the therapy of AF in clinics. We cannot clarify the exact compounds from SSYX that exert the protective function. Further study for exploring the molecular mechanisms underlying the anti-arrhythmic effect of exact bioactive components from SSYX in MetS rats would be necessary. Moreover, serves as an anti-arrhythmic agent, SSYX could also decrease the blood pressure in MetS rats, so it should be fully evaluated the safety effect of SSYX on the blood pressure in the further randomized controlled trial (RCT). In addition, sodium current (*I*_Na+_) is also potential anti-arrhythmic target. However, *I*_Na+_ was not evaluated in this study, further study focused on *I*_Na+_ should be assessed in the future.

## Conclusion

In conclusion, the present study provided novel insights that SSYX may exert anti-arrhythmia effect *via* increased *I*_to_, *I*_Ca-L_, and Cx43 expression through regulating TLR4/MyD88/CaMKII signaling pathway, which may be the mechanism of electrical remodeling MetS-induced VA.

## Data Availability Statement

The raw data supporting the conclusions of this article will be made available by the authors, without undue reservation, to any qualified researcher.

## Ethics Statement

The animal study was reviewed and approved by the Institutional Animal Care and Use Committee of Renmin Hospital of Wuhan University.

## Author Contributions

H-JY designed the study and wrote the paper. H-JY and J-JZ performed the experiments. H-JY, J-JZ, and WS prepared all figures and analyzed the results. BK and HH provided valuable suggestions and comments on the study design. All authors contributed to the article and approved the submitted version.

## Funding

This work was supported by grants from the key R&D Program (No. 2017YFC1700504), and the National Natural Science Foundation of China (No. 81570306).

## Conflict of Interest

The authors declare that the research was conducted in the absence of any commercial or financial relationships that could be construed as a potential conflict of interest.
