# Supplementary material for: Shensong Yangxin Protects Against Metabolic Syndrome-Induced Ventricular Arrhythmias by Inhibiting Electrical Remodeling
Source: Front Pharmacol. 2020 Jul 9;11:993. doi: 10.3389/fphar.2020.00993 (PMC7363804; doi:10.3389/fphar.2020.00993)
Supplement: Supplementary file 1 [file Image_1.pdf]

Supplementary Figure

Figure S1. Representative extracted ion chromatograms of the 10 analytes and Internal Standard (IS) in positive ion mode. 1, Morroniside; 2, Loganin; 3, Spinosin; 4, Paeoniflorin; 5, Coptisine; 6, Epiberberine; 7, Berberine; 8, Palmatine; 9, Schisantherin A; 10, Deoxyschizandrin; 11, IS. (A) Blank urine; (B) blank urine spiked with standard and IS; (C) rat urine after oral administration of Shensong Yangxin Capsule (SSYX).

Figure S1

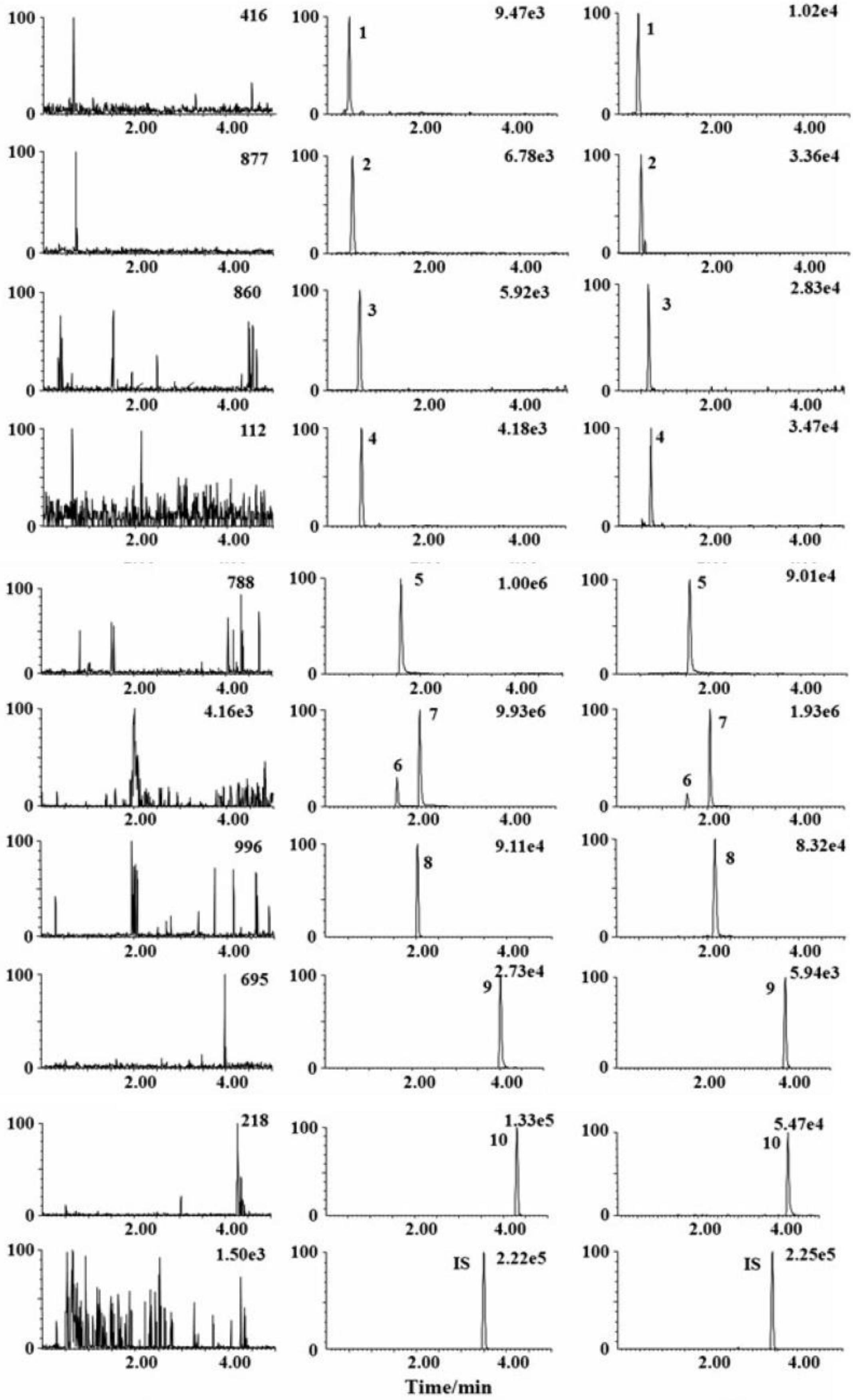

(Liu et al., *Biomed Chromatogr.* 2014; 28: 525-533)
