# Supplementary material for: Shensong Yangxin Protects Against Metabolic Syndrome-Induced Ventricular Arrhythmias by Inhibiting Electrical Remodeling
Source: Front Pharmacol. 2020 Jul 9;11:993. doi: 10.3389/fphar.2020.00993 (PMC7363804; doi:10.3389/fphar.2020.00993)
Supplement: Supplementary file 2 [file Table_1.docx]

**Supplementary Table S1.** The Chinese medcines contained in SSYX.

| Chinese name | Botanical name | The part used | Voucher numbers |
| --- | --- | --- | --- |
| Ren Shen | *Panax ginseng C.A. Mey.* | Root | 120103003 |
| Mai Dong | *Ophiopogon japonicus (Thunb.) Ker Gawl.* | Tuber | 120124056 |
| Shan Zhu Yu | *Cornus officinalis Siebold & Zucc.* | Fruit | 120124059 |
| Dan Shen | *Salvia miltiorrhiza Bunge* | Root | 120124053 |
| Gan Song | *Nardostachys grandiflora DC.* | Root | 120124054 |
| Sang Ji Sheng | *Taxillus sutchuenensis (Lecomte) Danser* | Stem | 120124058 |
| Suan Zao Ren | *Ziziphus jujuba Mill.* | Fruit | 120124007 |
| Chi Shao | *Paeonia lactiflora Pall.* | Root | 120101201 |
| Wu Wei Zi | *Schisandra sphenanthera Rehder & E.H. Wilson* | Fruit | 120124057 |
| Huang Lian | *Coptis chinensis Franch.* | Root | 120124055 |
| Tu Bie Chong | *Eupolyphaga seu Walker* | Whole animal | 120101201 |
| Long Gu | *Os Draconis* | Skeleton | 120124060 |
